# Using ChatGPT for human–computer interaction research: a primer

**DOI:** 10.1098/rsos.231053

**Published:** 2023-09-13

**Authors:** Wilbert Tabone, Joost de Winter

**Affiliations:** Department of Cognitive Robotics, Faculty of Mechanical, Maritime and Materials Engineering, Delft University of Technology, Delft 2628CD, The Netherlands

**Keywords:** prompt engineering, human-subject research, application programming interface (API), reproducibility

## Abstract

ChatGPT could serve as a tool for text analysis within the field of Human–Computer Interaction, though its validity requires investigation. This study applied ChatGPT to: (1) textbox questionnaire responses on nine augmented-reality interfaces, (2) interview data from participants who experienced these interfaces in a virtual simulator, and (3) transcribed think-aloud data of participants who viewed a real painting and its replica. Using a hierarchical approach, ChatGPT produced scores or summaries of text batches, which were then aggregated. Results showed that (1) ChatGPT generated sentiment scores of the interfaces that correlated extremely strongly (*r* > 0.99) with human rating scale outcomes and with a rule-based sentiment analysis method (criterion validity). Additionally, (2) by inputting automatically transcribed interviews to ChatGPT, it provided meaningful meta-summaries of the qualities of the interfaces (face validity). One meta-summary analysed in depth was found to have substantial but imperfect overlap with a content analysis conducted by an independent researcher (criterion validity). Finally, (3) ChatGPT's summary of the think-aloud data highlighted subtle differences between the real painting and the replica (face validity), a distinction corresponding with a keyword analysis (criterion validity). In conclusion, our research indicates that, with appropriate precautions, ChatGPT can be used as a valid tool for analysing text data.

## 1. Introduction

OpenAI's ChatGPT has taken the research world by storm, garnering media attention and sparking debates. Released in November 2022, ChatGPT has shown great promise in various areas, such as interpreting computer code [1,2], generating prompts for art-generating tools [3], document writing and translation [2], text interpretation and review [4,5], generating creatively written text such as poetry [6], writing hospital discharge summaries [7], and as an assistive tool in education [8–10], among many other applications. Although ChatGPT offers many opportunities [11], others have highlighted several risks, including the potential for plagiarism in academic writing [12–14] and education [15–18], generation of incorrect computer code [19] or incorrect mathematical solutions [20], and the possibility of inaccurate or biased output [21–24].

In order to better appreciate its functioning, it is useful to elucidate how ChatGPT was created [25]. ChatGPT is a machine learning model that has been designed and fine-tuned in several stages. The first is a pre-training stage where multiple terabytes of text data, which includes a general crawl of the Internet, including sources like Github, Wikipedia, and others, have been tokenized into numerical sequences. These sequences are processed through a transformer neural network, a model architecture proposed by Vaswani *et al*. [26] that uses self-attention mechanisms to understand the contextual relationship between words in a sentence. The transformer analyses the entire sequence of tokens at once rather than sequentially, making it effective in modelling complex patterns in the data. During the pre-training phase, the decrease in loss function corresponds to an improvement in the prediction accuracy of the subsequent word, a procedure demanding significant computational resources involving 1000s of GPUs over a period of months. The eventual base model effectively operates as a ‘next-word predictor’ and has a refined understanding of diverse concepts and textual structures. However, to function as a useful AI assistant, further refinements are implemented. In the supervised fine-tuning stage, human-created ‘ideal’ (i.e. helpful, truthful, harmless) responses to prompts are used to refine the model. Then, reinforcement learning from human feedback (RLHF) is applied. In the reward modelling stage, human workers rank multiple generated responses to prompts, to train the model to rank response quality. Finally, reinforcement learning is applied to the previous stage's reward model to refine the assistant model. The final product, ChatGPT, is a chat interface designed to function as a user-friendly assistant. An API is available as well, allowing researchers and practitioners to connect it to programming software and to set a number of parameters. One such parameter is the *temperature*, which controls the level of randomness in the output.

Despite its advanced training, GPT fundamentally differs from human information processing. ChatGPT is an autoregressive model, sequentially generating each new token predicated on the previously generated tokens within the same sequence [27]. It does not possess introspective capabilities, meaning it cannot self-correct or perform sanity checks on its outputs [28]. Furthermore, ChatGPT lacks deep reasoning abilities, and it cannot use tools like humans do, such as accessing Web information or calculators [11,20,29]. Several strategies can enhance ChatGPT's performance, such as prompt engineering to encourage more thoughtful responses by spreading reasoning across multiple prompts (‘chain of thought prompting’ [30,31]). Methods such as self-consistency [32], try-again [33], and backtracking [28] enable the model to review its output, and its capabilities can be enhanced by connecting it to tools like online apps or calculators [25,34–36].

Considering the immense popularity of the ChatGPT Web interface and API, there is a need for further research to ascertain how it can be used for scholarly purposes and to evaluate its validity in such contexts. In this work, we explored whether ChatGPT can be applied as a valid tool in Human–Computer Interaction (HCI) research. HCI is the field that focuses on the design and use of computer technology and examines interfaces between people and computers. HCI research may involve various types of research methods, including questionnaires, interviews, psychophysics methods, virtual reality setups, and field tests. In this process, the HCI researcher often collects text data. For example, in questionnaire research, a number of free-response items may be included to allow the respondents to reflect on a specific type of HCI design, while in interviews, verbal data is collected that is later transcribed and analysed [37–40], and in field or VR studies, a think-aloud protocol is often used to enrich the data collection [4,41–43]. The analysis of text-based data has faced criticism due to its perceived lack of reproducibility [44–46]. For example, despite the frequent use and acclaim for the thematic analysis method [47–49] in revealing underlying themes in texts, the consistency of themes identified by independent researchers analysing the same text, such as interview transcripts, remains a contentious issue [50,51].

This study posed the research question: To what extent does ChatGPT produce valid sentiment scores and summaries when applied to different forms of text data in HCI research? Our analysis was based on questionnaire responses, interviews, and think-aloud data available from three previous studies [52–54]. Our objective was to determine if ChatGPT's outputs could uphold both face validity and criterion validity. Here, face validity refers to plausibility—the extent to which ChatGPT appears, on face value, to accurately measure what it is supposed to. Conversely, criterion validity in our context refers to the extent to which the outputs of ChatGPT correlate with other established measures, namely automated or human-generated scores or counts. We conducted three distinct studies, using either the ChatGPT API (introduced on 1 March 2023) or the online website. Each study applied ChatGPT to different types of data (questionnaire data, interview data, and transcribed think-aloud data) to test its validity.

## 2. Study 1: questionnaire textbox data

**Figure 1.**
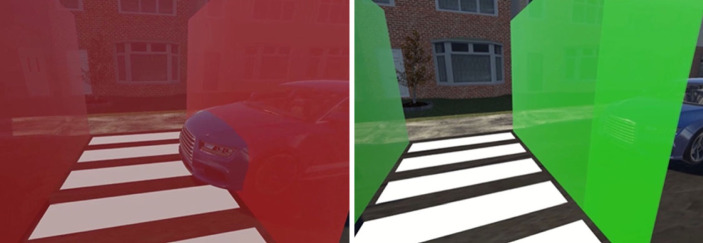
Frames from the videos presented in the online questionnaire. In this instance, the ‘Virtual fence’ (interface number 6) interface is seen in its non-yielding (left) and yielding states (right) [53].

In an online study by Tabone *et al*. [53], 992 respondents rated nine new augmented reality (AR) interfaces for pedestrian-vehicle interaction. Each interface was presented in two animated video clips depicting a crossing situation in a virtual environment, where a single vehicle approached from the right. The interface was presented in a yielding or non-yielding state, with the former communicating that the approaching vehicle would stop for the pedestrian and the latter communicating the opposite. Figure 1 provides still frames from the videos as an example. As part of the questionnaire, respondents had to complete several rating scales related to the interface's intuitiveness and convincingness in communicating the message to cross or not to cross the road. Rating scales regarding acceptance, attractiveness, aesthetics, ease of understanding, and the adequacy of information were also presented. A free-response item was added at the end, allowing respondents to elaborate on their ratings: ‘*Please add a few words to justify your choices above (*e.g. *comment on the shape, colour, functionality, and the clarity of the interface)*’.

In the original study, the 15 quantitative ratings for intuitiveness, convincingness, etc. were statistically analysed and averaged into a composite score, which was calibrated to have a grand mean of 0 and a standard deviation of 1. The meaning of the composite score can be characterized as ‘whether the AR interface is good or not’ [53].

With regard to the statistical reliability of the composite scores, Tabone *et al*. [53] reported that the Cronbach alpha was 0.990, as calculated across the matrix of 15 mean scores of the rating scales x 9 AR interfaces. We also determined the statistical reliability of the composite score using the split-half method. To this end, we repeatedly divided the 15 rating scale items into two groups of 8 and 7 random items, and calculated the composite score over these two sets separately. The two vectors of composite scores (9 × 1) thus obtained correlated strongly, on average, *r* = 0.995. Finally, we determined the composite score reliability by repeatedly splitting the group of 992 participants into two groups of 496 randomly selected participants. The two vectors of composite scores (9 × 1) that are thus obtained also correlated strongly on average, *r* = 0.992.

In the original study, the text-based responses were analysed through a manual review process where representative answers were selected. From the average 46 text comments selected per interface, four representative comments (two with positive sentiments and two with negative sentiments) were chosen for inclusion in the publication by Tabone *et al*. [53]. A limitation of this thematic analysis was the potential influence of the researcher's subjectivity on the analysis. Furthermore, the manual text analysis that we conducted had limitations in the sense that we could not easily compare or interpret the results in relation to other findings. More specifically, it was difficult to assess whether the results from our thematic analysis were somehow linked to the calculated composite score.

### 2.1. Methods

In the new analysis, all text responses were extracted from the raw questionnaire data, available from the online supplementary material of Tabone *et al*. [53]. This yielded 9 columns (1 for each interface) and 992 rows, for a total of 8928 entries. The data was unaltered, and the responses were submitted to the ChatGPT API through a custom script (date: 4 March 2023, model: gpt-3.5-turbo). The parameter governing randomness, referred to as ‘temperature’, was set to a value of 0. This configuration may be advantageous in tasks such as sentiment analysis, where the absence of random variation is preferred. This deterministic attribute of the API contrasts with the Web interface of ChatGPT, which features some randomness in its output.

The raw data submitted to ChatGPT included not only meaningful statements, but also contained meaningless ones, staccato text, and text in different languages (table 1 for a random selection of 20 comments for one of the nine interfaces). We have chosen to retain these occasionally meaningless quotes to evaluate ChatGPT's sentiment analysis in a standalone way, without human intervention. By not filtering the text, the sentiment analysis for ChatGPT becomes more challenging compared to a situation where a human researcher might preprocess the data; it falls to ChatGPT's model itself to handle gibberish text in an appropriate manner.

**Table 1.** Selection of comments about the ‘Virtual fence’ interface (20 of 992 comments were selected using a random number generator).

interesting and very good
the would be fun to test in the future
I have no idea
really clear, but a bit too much
full colour very clear
easy to see with the bright colours
best one so far...
not so great grapic quality
too big sign
I have no comment
on det kommer någon gående då stannar man eftersom det är det det betyder
extremely clear way to show when it is safe and unsafe to cross
big, bold and very clear
Bruce Springsteen is a communist
want to see by myself if it's safe
felt more intuitive than the second option but i still prefer the first option
the interface is too big
it is hard to trust
I think the fencing is a good way to signal whether or not it's safe to cross the street. However, the non-yielding fence isn't as effective at getting the message across, apart from the colour, as the pedestrian crossing is still open. I think it could be confusing, especially to someone who's colourblind. Maybe a big X to cross out the pedestrian crossing/obscure the path would be more effective here.
niet van toepassing
It was a bit confusing to show a crossing if you were not supposed to cross. Even though it was red I still saw the crossing and thought it was maybe OK.

Since there are limits to the amount of text (tokens) that could be submitted at any one time, it was decided to adopt a ‘hierarchical approach’ to data processing, where batches of text were submitted, and the numeric outputs were subsequently averaged. Specifically, each column was automatically divided into nine batches of 100 rows each, and a tenth batch consisting of 92 rows. Each batch was then separately submitted to the API, with a specific prompt:

‘The comments below were obtained from respondents in an online survey about an AR interface in a road-crossing scenario. If you read the comments below, what is the quality of the interface on a scale from 1 = bad to 10 = good? Only report a number between 1 and 10. Round to two decimals',

followed by the comments from each batch. After all the batches were submitted, the mean sentiment score for each interface was calculated by averaging the sentiment scores of its 10 batches. The correlation coefficient was computed between the mean sentiment score of the interfaces and the composite score of the interfaces as previously reported by Tabone *et al*. [53].

**Figure 2.**
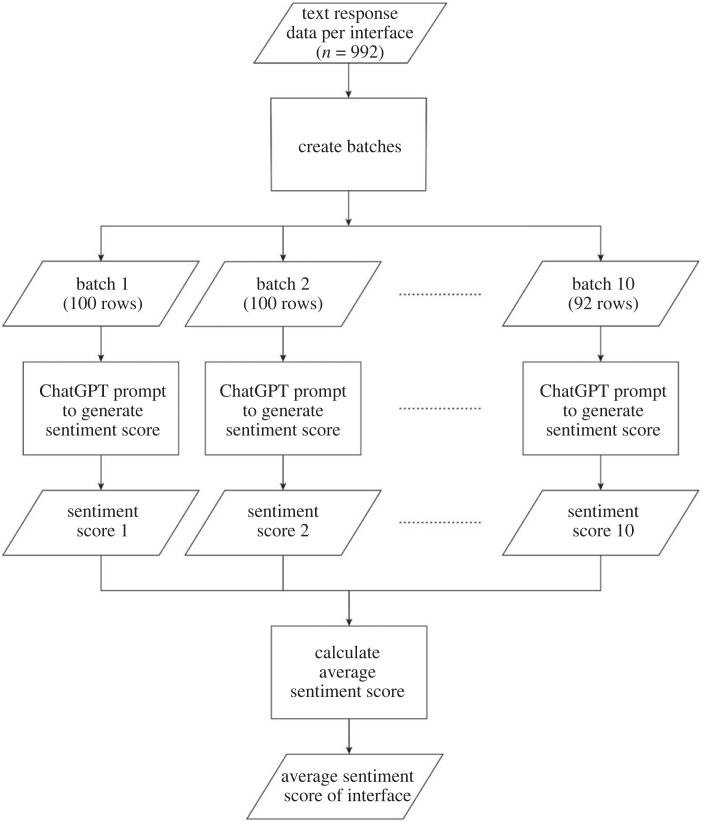
Flowchart detailing the process of the hierarchical approach used to calculate the average ChatGPT sentiment score per interface. The entire process was conducted nine times (once for each interface).

We opted to evaluate the correlation with the rating scales because these scales, which had been gathered from the respondents, provide a clear numerical criterion, enabling us to quantitatively assess the validity of ChatGPT's sentiment analysis. In the free-response item, participants were asked to elaborate on their previous answers to the rating scales. We therefore anticipate that if ChatGPT can produce a more meaningful sentiment score from the participants' text responses, there will be a stronger correlation (i.e. closer to 1.00) with the composite score. The entire process used to calculate the mean sentiment score is detailed in the flowchart presented in figure 2.

Next, we examined whether the prompt could be improved by trial and error. More specifically, we repeated the above process using different prompts, in an attempt to maximize the correlation coefficient between the mean sentiment score obtained through ChatGPT and the mean composite score [53].

A potential limitation of the above analysis is the calculation of correlations between the ChatGPT sentiment score based on textual responses and a numerical composite score grounded in rating scale responses. As previously indicated, we assume that a higher correlation coefficient is indicative of higher criterion validity. However, this supposition relies on the assumption that both the rating scales and textual responses have accurately captured the sentiment of the AR interface. Since this is not certain, we have substituted the composite score based on the rating scales with an alternative criterion, where we applied a traditional sentiment analysis method to the texts. To accomplish this, we first tokenized all the statements of the respondents for each AR interface. Subsequently, we subjected the tokens, for the 992 participants and nine AR interfaces separately, to the VADER (Valence Aware Dictionary and sEntiment Reasoner) algorithm [55]. VADER is a lexicon and rule-based sentiment analysis tool that quantifies the sentiment of text by assigning polarity scores to words. It generates a compound sentiment score ranging from −1 (most negative) to +1 (most positive), taking into account factors like negations, punctuation, capitalization, and emoticons. We then computed an average VADER sentiment score per AR interface by averaging over the 992 values, and correlated this VADER sentiment score with the ChatGPT sentiment score, which was based on the same text.

One limitation of ChatGPT we discovered is its peculiar preference for certain discrete values. These discrete outputs inherently have a negative effect on the sensitivity of the sentiment scores, especially when the number of batches is small. We also found that the order of the texts within a given batch had an impact on the output. These findings led us to realize that a refined method of sentiment analysis could be found in bootstrapping. To this end, for each of the nine AR interfaces, we drew a large number (200) of batches of 100 texts, randomly selected from the 992^1^. We submitted these batches in the same manner as above, and then took an average sentiment score over the outputs. We repeated this process for different prompts ranging from extensive prompts providing context about the survey in which videos were shown to respondents, to very short prompts, where the shortest prompt was ‘*Rate texts, from 1 to 100. Only report a single number between 1 and 100, rounded to two decimals. No text!:’*.

### 2.2. Results

The ChatGPT API provided outputs of the 90 batches (i.e. 9 interfaces x 10 batches per interface) in a total of 72 s on a standard laptop and Internet connection. The first prompt we tried (see Methods) yielded a very strong correlation between the ChatGPT outcomes and the composite from the numeric rating scales (*r* = 0.975). In other words, ChatGPT produced sentiment scores that correlated extremely highly with aggregated human ratings.

**Figure 3.**
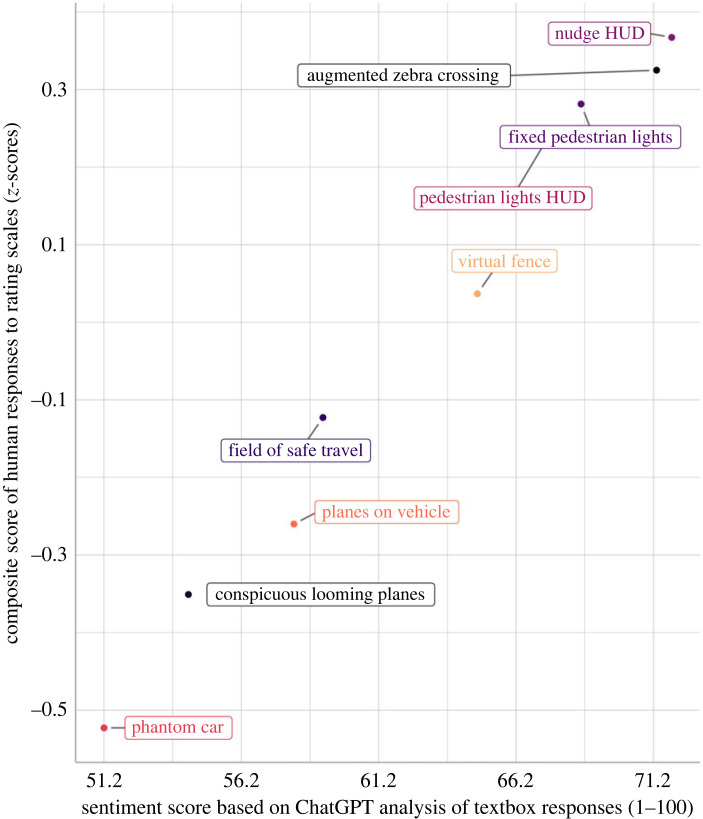
Scatter plot showing the composite score based on rating scales in the online questionnaire study (extracted from [53]) against the mean sentiment score calculated from the ChatGPT outputs, for nine augmented reality interfaces. The composite score has a mean of 0 and a standard deviation of 1 in the population of respondents.

Through trial and error, we modified the prompt as follows: ‘*Looking at the comments, score the interface, from 1 to 100. Only report a number between 1 and 100, rounded to two decimals*'. The correlation coefficient between the two measures was found to be close to 1 (*r* = 0.993). The scatter plot in figure 3 shows the mean sentiment score from the ChatGPT outputs and the composite score based on the rating scales.

**Figure 4.**
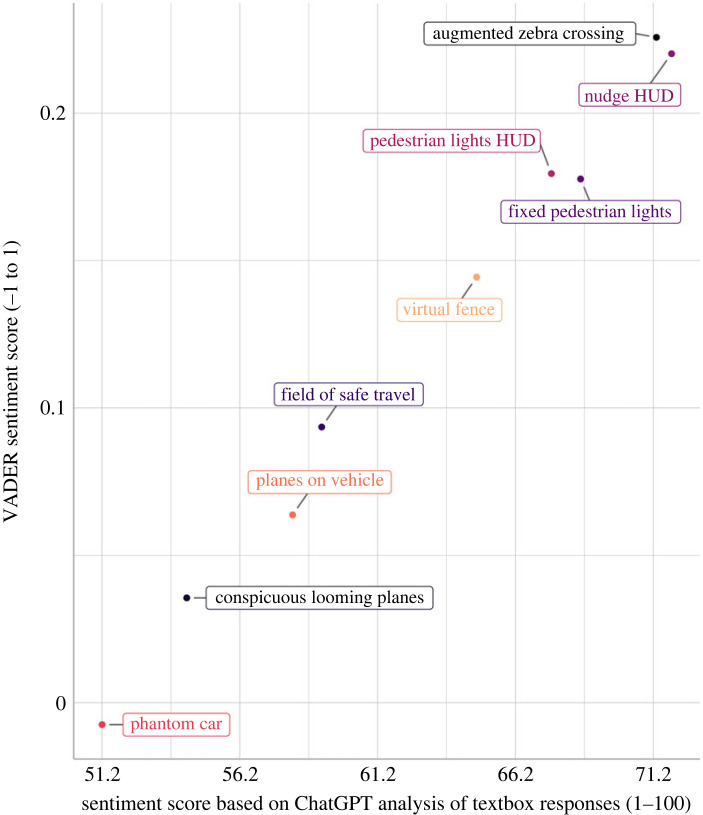
Scatter plot showing the sentiment score calculated using the VADER (Valence Aware Dictionary and sentiment Reasoner) sentiment method, a lexicon and rule-based algorithm [55] against the mean sentiment score calculated from the ChatGPT outputs, for nine augmented reality interfaces.

The ChatGPT sentiment score not only strongly correlated with the composite score as calculated from the rating scales completed by the same participants; it also correlated strongly with the VADER sentiment score, a more classical lexicon rule-based method, specifically with *r* = 0.996. This correlation coefficient is illustrated in figure 4. The VADER sentiment score also strongly correlated with the composite score (*r* = 0.992).

It is noteworthy that the prompt used is short, and devoid of context. During the trial-and-error process, we discovered that longer prompts, which provided more context (such as that the experiment involved AR and a car and pedestrian in a road crossing scenario) were not needed to achieve a high correlation. It was also not necessary to mention the dimension across which the sentiment score should be provided (e.g. quality, clarity).

We also explored the impact of batch size and the randomness parameter on sentiment analysis results. We found that using a smaller batch size (of 25 instead of 100 comments) did not substantially affect the correlation coefficient; it changed from 0.993 to 0.987. We also tried the alternative, namely submitting all 992 comments per AR interface at once, making use of a recent model of ChatGPT that can process 16 384 tokens (gpt-3.5-turbo-16k-0613). This approach turned out to be less effective, resulting in a correlation coefficient between the output and the composite score of only 0.724.

**Figure 5.**
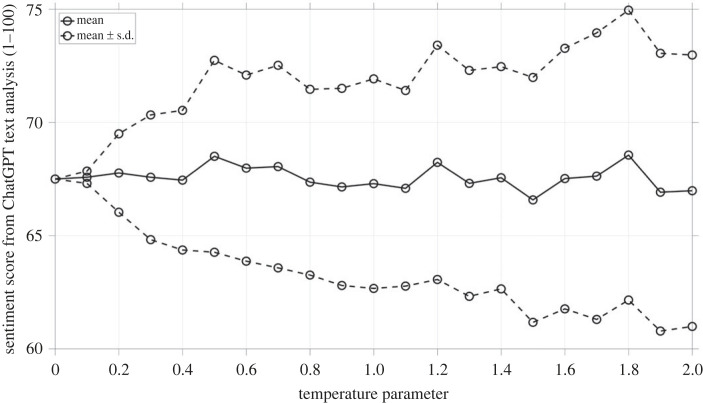
Mean and mean ± standard deviation of the sentiment score based on ChatGPT analysis of textbox responses, for one batch of texts (*Augmented zebra crossing*, texts 1–100 of 992), as a function of the temperature parameter (in increments of 0.1).

Furthermore, we found that adjusting the randomness parameter (by setting it to a higher value than 0) and averaging the results of multiple repetitions did not increase the correlation coefficient either. Figure 5 provides an illustration of this finding. We generated 100 sentiment scores for the first batch of comments, specifying the number of scores by setting the ‘n’ parameter to 100 in the API request. We repeated this process for different ‘temperature’ settings, from 0 up to the maximum possible value of 2. We then calculated the mean and standard deviation across these sentiment scores. As shown in figure 5, the mean remained relatively constant across the different temperature settings. Conversely, the standard deviation grew with higher temperature values. This indicates that the sentiment analysis output remained unbiased irrespective of the temperature setting used. In other words, in the context of this sentiment analysis task, there seems to be no specific need for a nonzero temperature setting.

As mentioned, using the initial prompt yielded a strong correlation of 0.975 between ChatGPT's sentiment analysis and the average human scores. By modifying the prompt, we were able to increase this correlation to 0.993. However, one limitation was that the output was of limited resolution. To illustrate, of the 90 outputs (9 AR interfaces × 10 batches per AR interface), the score ‘67.50’ occurred 18 times, the score ‘62.50’ 13 times, and the score ‘54.29’ 6 times. A subsequent question we addressed is whether the correlation coefficient could be further improved through bootstrapping. Using the bootstrapping method, after trying 24 different prompts, we were able to increase the correlation coefficient to 0.997, with the following prompt: ‘*These text messages are obtained from a textbox in a questionnaire about nine interfaces for pedestrians who interact with an automated vehicle. I need you to provide a single sentiment rating about the interface being discussed in the comments, from 1 to 100. Only report a single number between 1 and 100, rounded to two decimals. No text!:*’ It was also found that overly truncating the prompt did not help; the shortest prompt mentioned above resulted in a correlation of 0.984.

In summary, it was found to be possible to obtain a near-perfect association between text sentiment scores obtained with ChatGPT, on the one hand, and numerical sentiment scores obtained using human responses to rating scales and lexicon and rule-based sentiment scores, on the other.

## 3. Study 2: interview data

**Figure 6.**
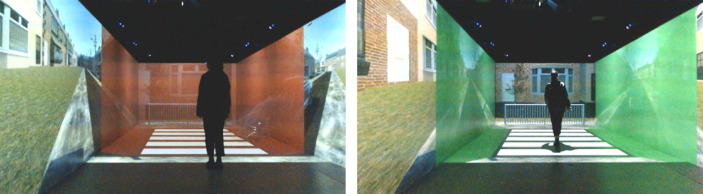
Non-yielding (left) and yielding (right) state of the *Virtual fence* interface.

In Tabone *et al*. [54], the effect of nine AR interfaces, as well as a control condition without AR interface, on pedestrian crossing behaviour was investigated in a virtual simulator environment. This was a follow-up of the online study described in the previous section and was conducted at the University of Leeds using the Highly Immersive Kinematic Experimental Research (HIKER) simulator (figure 6). The experiment was approved by the University of Leeds Research Ethics Committee under ethics reference number LLTRAN-150. All participants provided written informed consent.

The experiment consisted of 120 trials per participant, each featuring one of the interfaces, a different location of a visual attention attractor, and a vehicle which could either be yielding or non-yielding. The 120 trials followed a counterbalanced blocked design, divided into 10 blocks (with each block corresponding to one interface). After each block, the participant was interviewed for about 3 min, starting by asking whether they were comfortable, followed by, ‘*what did you think of this particular interface/situation?*’. Depending on the participant's response, follow-up questions about the clarity of the colour coding (e.g. ‘*is the colour code making any sense to you?*’) and a comparison between different interface states (e.g. ‘*between the two states, were there any preferences?*’) were asked.

Processing the interview data would be a challenging task, involving considerable transcription work and subjective interpretation. Therefore, it was decided to transcribe the audio-recorded interviews automatically and submit the transcripts to ChatGPT. In this paper, we examine whether ChatGPT provided valid insights into the qualities of each interface.

### 3.1. Methods

**Figure 7.**
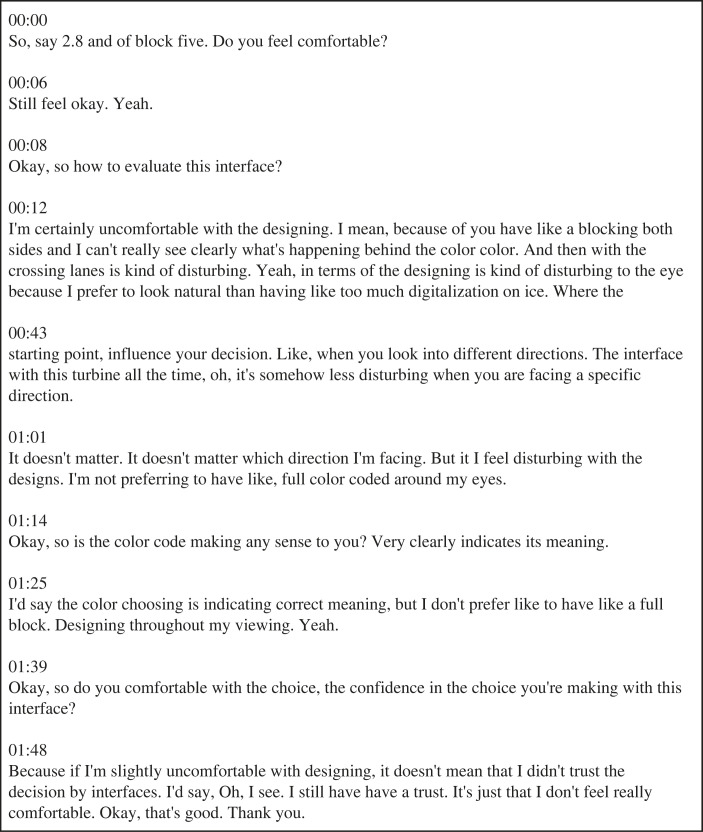
Example of an automated transcription (Participant 8, *Virtual fence*), created by Otter.ai [56].

First, the 300 voice recordings were transcribed by submitting the audio files to the paid online service Otter.ai [56]. An example of a transcript for the *Virtual fence* is shown in figure 7. As can be seen, the transcript did not distinguish between the interviewer and interviewee, and contained some transcription errors.

Next, each anonymous transcript was submitted to the ChatGPT API (date: 4 March 2023, model: gpt-3.5-turbo) using a custom script. The following prompt was used: ‘*Looking at the participant's responses, provide a summary of the interface*’, followed by the participant's interview on the subsequent lines.

**Figure 8.**
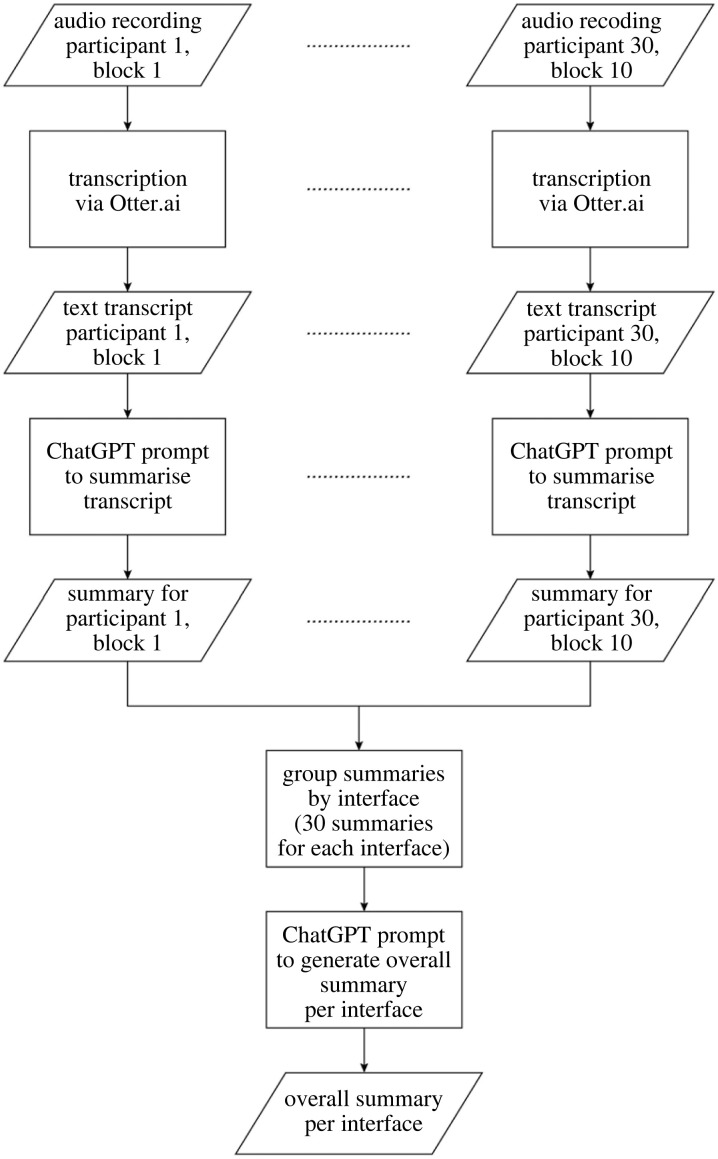
Flowchart detailing the process of transcribing and summarizing each transcript. Transcripts were then grouped per interface, and an overall summary was generated using a further ChatGPT prompt.

After collecting all responses from all 300 interviews, the responses per interface were again submitted to ChatGPT. The same prompt was used: ‘*Looking at the participant's responses, provide a summary of the interface*’, including all 30 summaries from the participants. The entire process used is detailed in the flowchart presented in figure 8.

A key challenge in assessing the validity of interview summaries stems from ChatGPT's tendency to always generate a plausible output. Deciding if the output is truth-based (i.e. valid) or a hallucination (a known issue with large language models; [57]) requires further consideration. We have chosen to evaluate the validity of one interview summary by involving an independent human rater. A fellow researcher (not an author) was provided with the 30 interview transcripts for one of the interfaces (*Virtual fence*), with the task of performing a content analysis. This fellow researcher created categories (e.g. ‘Interface intuitive’, ‘Walls gave a safe feeling’), split the transcript into individual sentences or sub-sentences expressing a single notion, and classified each of these into one of the categories. The researcher adopted an iterative procedure: as more transcripts were examined, categories were added and refined to capture emerging categories.

Upon completion of this independent analysis, the authors quantified the frequency of each category's occurrence by counting how many of the 30 participants made a remark per category. If the same participant mentioned a topic for a category more than once, this was counted as one instance for the category. Subsequently, we evaluated whether the summary generated by ChatGPT corresponded with the counts obtained by the fellow researcher.

Lastly, we evaluated whether ChatGPT4, OpenAI's paid version that outperforms ChatGPT3.5 on various benchmark tests [11,58,59], would yield a richer and more accurate summary. To do this, we repeated the aforementioned process, again via the API, but this time using the gpt-4-0613 model.

### 3.2. Results

The result was a summary of the qualities of each interface (see [54], for all summaries). Our script, which connected to the ChatGPT3.5 API, took a total of 25 min to process all 300 interviews, including creating the overall summaries. The overall summary for the *Virtual fence* is provided below:

‘Overall, the participants had mixed feelings about the interface. Some found it clear and easy to use, while others found it confusing and overwhelming. The green and red states were generally well-received, with participants feeling safer and more confident when the green state was active. However, some participants had concerns about the interface obscuring their view of the road and potentially causing accidents. The zebra crossing was generally appreciated, but some participants found it confusing and potentially misleading. Accessibility features were also noted as a positive aspect of the interface. Overall, there were varying opinions on the effectiveness and usability of the interface.’

The results of the content analysis, shown in table 2, are generally in line with the above summary of the interviews. For example, the content analysis indicated that 8 participants stated that the tunnel walls inhibited visibility, and 3 participants suggested that the walls provided a false sense of security, which relates to the summary statement: ‘*… some participants had concerns about the interface obscuring their view of the road and potentially causing accidents.’* The remark about the zebra being potentially misleading was noted by 2 participants in the content analysis (‘*Red is counterintuitive because it shows a zebra’*), which aligns with the summary (‘*…some participants found it confusing and potentially misleading.’)*. The overall result of ‘mixed feelings’ is also in alignment with the content analysis, where some participants found the tunnel intuitive, but others found it confusing.

**Table 2.** Results of the human content analysis of the 30 interviews. Only topics mentioned by at least two participants are mentioned in this table.

experimenter questions	number of participants
experimenter - wellbeing monitoring	26
experimenter - thoughts on interface	26
experimenter - preference between interfaces	20
experimenter - clarification/follow-up question	19
participant responses	number of participants
no preference between green and red	10
walls inhibited visibility	8
relied on the interface more than the car/interface influenced decision	8
walls gave a safe feeling	8
interface clear	7
interface intuitive	7
green preferred	7
interface not intuitive/counterintuitive/confusing	5
red preferred	5
interface salient/large	4
interface safe	4
relied on the car more than the interface/interface did not affect decision	4
one trial needed for understanding	3
interface easy	3
interface too large	3
red not intuitive/counterintuitive/confusing	3
interface not safe	3
walls provided false safety	3
interface had too many signals/too much going on	3
bad that interface did not explicitly relate to the approaching car	3
felt confident	3
realistic	3
unclear/gibberish	3
green intuitive	2
red is counterintuitive because it shows a zebra	2
good that the interface was not attached to the approaching car	2
liked the interface	2
did not feel comfortable with the interface	2

The final summary using ChatGPT4 is provided below:

‘*The participants' responses to the interface were mixed. Some found it intuitive and easy to understand, appreciating the clear signals of when to cross and when not to cross. They felt safer and more confident when using the interface, particularly appreciating the green signal that indicated it was safe to cross. However, some participants found the interface confusing and overwhelming, particularly in relation to the green and red signals. They felt that the interface was too busy and cluttered, and that the colors could be confusing, especially for colorblind individuals. Some participants also expressed concern about the interface potentially impacting their safety due to its high walls and lack of transparency, which could obscure their view of other vehicles or cyclists. Despite these concerns, most participants were able to adapt to the interface by the end of the session and found it generally helpful in making crossing decisions. Some participants suggested improvements, such as adding audio support or making the interface more natural-looking rather than fully digitalized*.’

It can be seen that the ChatGPT4 summary is richer and touches on more facets than the ChatGPT3.5 summary. The ChatGPT4 summary makes the point that the interface was too overwhelming or cluttered, which coincides with the content analysis, in which 4 participants mentioned that the interface was large, 3 found it too large, and 3 said there ‘too much going on’. The summary also offers recommendations on how the interface can be improved. One point of criticism is that the ChatGPT4 summary mentions issues with audio support and colour blindness, while these topics did not emerge in the content analysis. Our inspection of the transcripts mentioned the word ‘colorblind’ only once, and the word ‘audio’ (or ‘sound’) also only once.

## 4. Study 3: think-aloud data

In this study, data from a think-aloud protocol was recorded while participants, wearing a mobile eye tracker, freely gazed at Rembrandt's *The Night Watch* (1642) in Amsterdam's Rijksmuseum [52]. Unlike Studies 1 and 2, Study 3 does not involve HCI; participants expressed their thoughts through verbalization while examining a painting, rather than interacting with a computerized system. Nevertheless, the use of the think-aloud method is firmly rooted [60,61] and often employed [62–64] in the HCI domain, and can therefore be viewed as a prototypical tool within this field.

**Figure 9.**
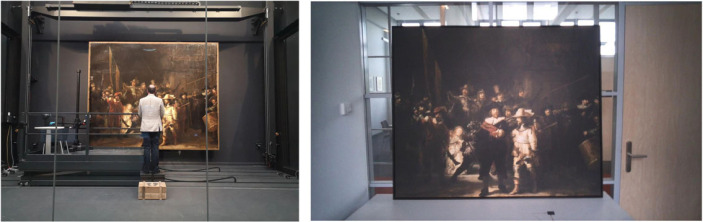
Experimental setup for *The Night Watch* study in a glass chamber in the Rijksmuseum (left) and a replica in an office environment (right) [52].

The aim of the original study was primarily to assess the participants' attentional distribution for this work of art. The analysis of the gaze data was supported by the recorded statements from the participants as they freely spoke out their thoughts while gazing at the painting (figure 9, left).

The recorded voice data (in Dutch) was previously transcribed and analysed using a hybrid approach, combining thematic analysis with a quantitative method of tabulating word frequencies. The main themes were identified, and the number of times a target word belonging to each theme was spoken was also counted.

A follow-up experiment was conducted to assess the robustness of the first experiment, using a replica of the painting in a laboratory setting and with new participants (figure 9, right). The participants in the Rijksmuseum study were recruited by contacting acquaintances of the authors and a television crew and by inviting (ex-) students, whereas participants in the replica study were recruited from the student population and the teaching and administration staff of the Delft University of Technology. The task instructions and experiment duration (5 min of viewing per participant) were identical between the two settings.

The authors discovered that admiration-related words (i.e. beautiful, cool, enjoy, fantastic, fascinating, great, impressive, nice, special, splendid, unique, wonderful, wow) were uttered more frequently for the real painting compared to the replica painting; other comparisons of think-aloud data between the two versions of the painting did not yield statistically significant differences [52].

### 4.1. Methods

The supplementary material for *The Night Watch* experiment [52] contains two transcript files in Dutch, one for participants observing the real painting in the Rijksmuseum, and another for participants looking at the replica painting in the laboratory.

A pairwise comparison was conducted between transcripts by order of participant numbers, i.e. the first transcript in one file was compared to the first transcript in the other, and so on. Since there were a total of 21 participant transcripts in the recordings taken at the Rijksmuseum, and 27 for the replica, the new analysis included only the first 21 participant pairs.

We used the online chat window, instead of the API used in Studies 1 and 2. On January 16, 2023, each participant pair was compared in a separate chat window, using three prompts. The first prompt was: ‘*Consider the following transcripts:*’, followed by the transcript of the original painting. Based on this prompt, ChatGPT automatically provided a short summary in English. This was followed by a transcript related to the original painting: ‘*How does it differ from the following transcripts, in essence?*’, followed by a transcript from the replica painting. Based on this prompt, ChatGPT produced a comparison of the two transcripts. Next, the following prompt was given: ‘*What would you regard as the key difference between both transcripts?*’

**Figure 10.**
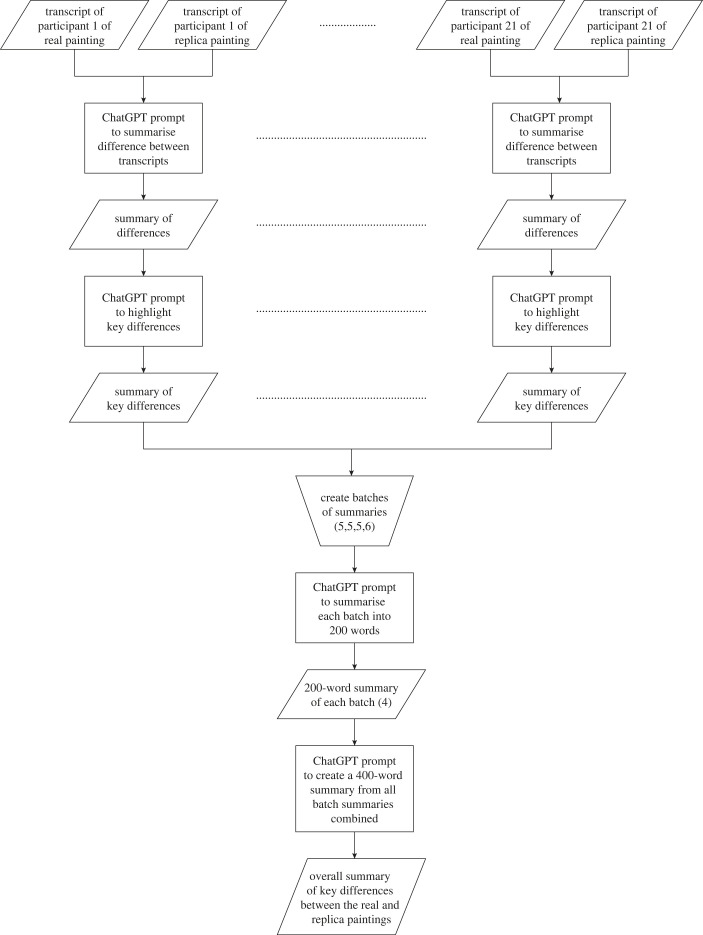
Flowchart detailing the process used to summarize the key differences between the real and replica *The Night Watch,* as stated by participants in a think-aloud protocol.

To arrive at the final output, a hierarchical approach was again adopted. Specifically, once all 21 comparisons were performed, the key difference outputs were grouped into batches of 5, 5, 5 and 6. Each batch was summarized into a 200-word paragraph using the prompt ‘*Please provide a 200-word summary for the following text:*’. The four resulting summaries were then combined and condensed into a general 400-word summary using the prompt: ‘*Please provide a 400-word summary for the following text:*’. The entire process used is detailed in the flowchart presented in figure 10.

In Study 1, the sequence in which texts were submitted to ChatGPT was found to have some influence on the obtained sentiment score, even when the content of the texts remained the same. In addition to the fact that ChatGPT has a token limit, this sensitivity to the prompt was a reason for us to submit the text in batches and subsequently average the sentiment scores. A similar limitation might affect the aforementioned analysis, where we entered the transcripts corresponding to the real *The Night Watch* painting first, followed by those pertaining to the replica. To investigate whether this sequence introduces a potential bias, we performed an additional analysis through the API (model: gpt-3.5-turbo-0613). Our employed method is more comprehensive than the approach executed with ChatGPT via the Web interface and involved prompting ChatGPT as follows: ‘*TRANSCRIPT 1: <transcript of a participant for the real painting> TRANSCRIPT 2: <transcript of a participant for the replica painting> What would you regard as the key difference between both transcripts?*’

This procedure was conducted for all 567 possible pairs, combining participants from the real painting (21 participants) with those from the replica painting (27 participants). The 567 pairs were sorted in random order. We then repeated this process but for the real painting and the replica submitted in reverse order. Accordingly, we prompted ChatGPT as follows: ‘*TRANSCRIPT 1: <transcript of a participant for the replica painting> TRANSCRIPT 2: <transcript of a participant for the real painting> What would you regard as the key difference between both transcripts?*’. Consequently, we obtained two sets of 567 descriptions of how the transcripts for the replica and real *The Night Watch* differed from one another, once for the situation where the real painting was first mentioned in the prompt, and another time where the replica was first mentioned in the prompt.

Subsequently, we implemented the following procedure for each sequence to obtain a final summary. First, we separated the 567 pairwise comparisons into 7 batches of 81 each, maintaining their original random order. We then submitted each batch to the ChatGPT API (model: gpt-3.5-turbo-16k) using the following prompt: ‘*These are transcripts of different pairs of speakers. Provide one single 200-word summary of all Transcripts 1 combined; as well as one single 200-word summary of all Transcripts 2 combined. Report as follows: ‘Transcript 1: …; Transcript 2: …*’’. Lastly, for both sequences, we executed a prompt to create a final summary, using the following prompt: ‘*Provide one single 200-word summary of the differences between Transcripts 1 and 2, of all batches combined:*’, followed by the summaries of the 7 batches. The objective of this analysis was to determine whether the final summaries were consistent, irrespective of whether the real painting or the replica painting was presented first in the prompt.

### 4.2. Results

The text below shows the final 400-word summary that was generated by ChatGPT, highlighting the differences between what participants said while observing *The Night Watch* in the museum and its replica in the laboratory:

‘The text describes the differences between two transcripts of two people's observations of a painting. The first transcript is more emotive, focusing on the personal experience of looking at the painting, showing awe and amazement, trying to understand the meaning, and expressing it as a once in a lifetime experience. The person in this transcript is deeply moved by the painting and finds it fascinating, providing a detailed description of the painting and its intricate details, dynamic lighting and highlights on the faces, and expressing its beauty and realism.

The second transcript is more analytical, providing a more general and surface-level description of the painting, focusing primarily on the activity of the figures and their presence of weapons and musical instruments. It also notes the lighting, but makes less interpretive statements and has less focus on the individual figures and their meaning in the scene. Additionally, the second speaker seems to have a better knowledge of the painting and its historical context, including information on its restoration and the identification of specific elements such as a dog, chicken or dove, and the presence of Rembrandt himself in the painting.

The main difference between the two transcripts is the tone, focus, and level of detail provided by the speakers. The first transcript is more emotional and expresses awe and wonder towards the artwork, while the second transcript is more analytical, providing objective observations and less emotive language. Additionally, the second speaker seems to be more knowledgeable about the painting and its historical context, but less engaged with the painting, providing a more general and surface-level description of the painting.’

The generated summary above indicates that ChatGPT captured how the participants looking at the real painting were ‘deeply moved by the painting’, and it commented on the ‘dynamic lighting and highlights on the faces’. Moreover, the participants who viewed the real painting were deemed to be ‘more emotional’ and expressed ‘awe and wonder towards the artwork’, in contrast to the participants who viewed the replica, which were described as being ‘more analytical, providing objective observations and less emotive language’.

The aforementioned analysis was conducted using the ChatGPT Web interface for 21 paired comparisons. Our more refined analysis, in which all 567 possible pairs between participants were reduced to final summaries through the API, resulted in the following final summary for the situation where the transcripts for the real painting (Transcript 1) were presented before the transcripts of the replica painting (Transcript 2):

‘The key difference between all the transcripts in the combined batches is the level of detail, analysis, and engagement provided by the speakers. Transcript 1 consistently offers more detailed descriptions and observations of the painting, focusing on elements such as lighting, composition, colors, clothing, and specific details. The speakers in Transcript 1 express fascination, admiration, and curiosity about the painting, providing personal insights, interpretations, and emotional responses. They also discuss the historical context and significance of the artwork. In contrast, Transcript 2 consistently provides more general and superficial descriptions, focusing on the overall impression, recognition of figures, and some specific details. The speakers in Transcript 2 express uncertainty, confusion, and a lack of knowledge or familiarity with the painting, often speculating on possible meanings and interpretations. They do not provide as much detail, analysis, or personal engagement as the speakers in Transcript 1. Overall, Transcript 1 demonstrates a deeper level of engagement, understanding, and appreciation for the artwork, while Transcript 2 offers a more casual and less informed perspective.’

For the reversed situation, where the transcripts for the replica painting were submitted first (Transcript 1) followed by the transcripts for the real painting (Transcript 2), the final summary was as follows:

‘The key difference between the transcripts in all batches combined is the level of detail and analysis provided. Transcript 1 tends to provide more general and surface-level descriptions of the painting, focusing on the overall scene, main figures, and notable details. The speakers in Transcript 1 also express uncertainty and lack of knowledge about the historical context. In contrast, Transcript 2 demonstrates a deeper appreciation and engagement with the painting. The speakers in Transcript 2 mention specific details they find fascinating, such as the eyes, hidden elements, and clothing. They also reflect on their personal experiences and emotions while viewing the painting. Transcript 2 provides a more in-depth observation and analysis of the artwork, discussing elements such as composition, lighting, colors, and interactions between figures. The speakers in Transcript 2 also question the authenticity and meaning of certain elements, providing a more analytical and subjective response to the painting. Overall, Transcript 2 offers a more detailed and analytical description of the painting, while Transcript 1 tends to provide more general and observational descriptions.’

It can be seen that the two summaries above for the real painting have several similarities. Both refer to detailed descriptions and observations of the painting, with emphasis on composition, lighting, and colours. They also show an emotional and personal connection to the artwork, discussing their feelings and insights (‘fascination, admiration, and curiosity about the painting, providing personal insights, interpretations, and emotional responses’ versus ‘details they find fascinating, such as the eyes, hidden elements, and clothing. They also reflect on their personal experiences and emotions while viewing the painting’). Additionally, the speakers touch upon the deeper meanings, significance, or questions related to the artwork (‘discuss the historical context and significance of the artwork’ versus ‘question the authenticity and meaning of certain elements, providing a more analytical and subjective response to the painting’).

With regard to the replica, both summaries mention providing more general descriptions (‘more general and superficial descriptions’ versus ‘more general and surface-level descriptions’). The summaries also highlight a focus on the overall impression (‘overall impression’ versus ‘overall scene’) and the key figures (‘recognition of figures’ versus ‘main figures’), and both emphasize the speakers’ ‘uncertainty’ and ‘lack of knowledge’ in their discussions about the painting.

In conclusion, the final summaries are robust across prompting sequences (real painting first versus real painting second) and in line with the keyword analysis by De Winter *et al*. [52] which found more admiration for the real painting compared to the replica. Simultaneously, it can be observed that the two summaries pertaining to the transcripts of the real painting and the two pertaining to the replica exhibit differences in their specific phrasings, even though they originate from the same transcripts and the only difference was the sequence of prompting. The full analysis can be found in the supplementary material, and the findings are further discussed in the Discussion section.

## 5. Discussion

In this study, we examined whether ChatGPT can extract valid patterns from text data. Firstly (Study 1), we had ChatGPT perform a sentiment analysis on texts from a questionnaire study, and assessed criterion validity by calculating the correlation coefficient with average numeric rating scale scores of AR interfaces as well as with a lexicon and rule-based sentiment analysis method. Secondly (Study 2), we had ChatGPT summarize raw interview transcripts without preprocessing, and assessed face validity by evaluating the summaries, as well as criterion validity by comparing it with the results of a manual content analysis. Thirdly (Study 3), we examined whether ChatGPT is powerful enough to detect differences between think-aloud transcripts related to two different experimental conditions, namely a real painting versus a replica. Here, we assessed face validity by seeing if the summaries were sensible, as well as criterion validity by comparing the summaries with the results of prior keyword counts.

In the evaluation of questionnaire textbox responses (Study 1), ChatGPT exhibited remarkable proficiency in assessing sentiment. This was demonstrated by the virtually perfect correlation (*r* > 0.99) between the ChatGPT sentiment scores and the pre-existing scores derived from both rating scales. This correlation was on a par with the statistical reliability of the criterion rating scales themselves, effectively reaching the upper ceiling of statistical similarity. The correlation between the ChatGPT sentiment score and the scores obtained through a traditional lexicon and rule-based sentiment analysis (VADER) was nearly perfect as well (*r* = 0.996). Collectively, these results indicate that ChatGPT possesses criterion validity in sentiment analysis with respect to numerical responses from human participants, as well as in relation to a previously published popular method of sentiment analysis. The strength of ChatGPT lies in its generalizability; whereas the VADER method has been specifically developed for sentiment analysis, ChatGPT is not, which highlights its potential for different types of numerical analyses of text data.

We also demonstrated several techniques that can be used to improve the criterion validity coefficient, namely, adjusting the prompt so that it contains sufficient (but not more than needed) context and the use of a bootstrapping method. The essence of the bootstrapping method is that text comments are repeatedly submitted to ChatGPT in random order, averaging out the effect of ChatGPT's preference for certain discrete numerical outputs as well as its sensitivity to the order in which texts are submitted.

In Study 2, ChatGPT was found to be a valid tool for summarizing interview data from human subjects. The interview summary was found to be logically interpretable and largely corresponded with the results of a content analysis conducted by a human researcher who manually tabulated the interview results. In our case, we found that ChatGPT3.5 provided a basic yet accurate summary, while ChatGPT4 offered a more refined summary of the interviews. However, the ChatGPT4 summary also included some points which were perhaps considerate and relevant, but not main themes in the raw transcripts. Another topic from the content analysis, namely whether the pedestrian based the crossing decision on the car or the AR interface, did not appear in either of the two ChatGPT summaries. A possible explanation is the hierarchical summarization method we used, which caused less essential yet recurrent information to fall away in the intermediate steps.

Finally, in Study 3, ChatGPT was found capable of highlighting subtle differences in what participants said when observing *The Night Watch* in the Rijksmuseum compared to a replica in an office environment. These results agree with the manual analysis of the think-aloud method, which revealed that the participants uttered more words related to admiration for the real *The Night Watch* than for the replica. Our final summary also adds to the ongoing discourse surrounding the effects of a painting in the context of the museum [13,65,66] and in a laboratory setting [67]. However, unlike the manual analysis performed by De Winter *et al*. [52], which provided keywords grouped by theme, ChatGPT was able to generate a paragraph that highlighted differences between the major themes identified in the two settings by submitting transcripts without further context.

It is important to note that if a single human researcher were to have generated the text output for either Study 1, 2, or 3, it may not be deemed trustworthy from a scientific perspective due to the limitations of human cognition, such as confirmation bias. It is possible that a human researcher, especially after having invested substantial effort into conducting the experiments, may expect or hope that certain differences between the two settings arise based on preconceptions or conflicts of interest. ChatGPT, having been provided with no prior information regarding the authenticity of the two paintings, created a discerning meta-summary. This approach, which is in alignment with the principles of researcher blinding, instils a certain level of confidence. However, as shown in Study 2, outputs from ChatGPT should not be unconditionally trusted. The value of ChatGPT may lie in pairing its outputs with other forms of analysis, such as counts and manual content analysis. Moreover, ChatGPT could serve as an auxiliary verification of human analyses, especially when there is a limited number of human annotators where, for example, an inter-rater reliability score cannot be calculated.

Apart from the validity assessments, it is noteworthy that the use of ChatGPT was time-efficient. Using the method presented in this paper, for Study 1 and 2, our script produced outputs in about 1 min and 25 min, respectively, while for Study 3, it only required sorting the data and copying it into the ChatGPT interface. An additional benefit of our pipeline was that the interview data of Study 2 was transcribed automatically, significantly reducing the time required compared to manual transcription. By contrast, the manual analysis of interviews can be challenging, particularly when dealing with large amounts of data, and subjective biases of the researcher may potentially influence the interpretation of the responses [68,69]. At the same time, it must be acknowledged that the development of the pipelines (as shown in the flowcharts in this paper) and the writing of the scripts that interacted with the API required a substantial amount of the researchers' time, scripts that are available for reuse (see supplementary material).

We also found that, although it is commonly advised that ChatGPT and similar large language models require prompts that provide suitable context (e.g. [70,71]), we found that brief prompts were sufficient for performing a sentiment analysis. We also found that the randomness (temperature) parameter should be set to 0 for accurate results.

When researchers wish to use ChatGPT, several limitations must be taken into consideration. Firstly, while ChatGPT offers high reproducibility when the temperature parameter is set to 0, it is crucial to understand that a reproducible output does not guarantee robustness. As pointed out above, ChatGPT is an autoregressive model, which implies that each produced token relies on the preceding prompt and the tokens generated up to that point. This dependency implies that, while ChatGPT's output is highly reproducible, it can be sensitive to small changes to the prompt (see also [11,72]) and differently fine-tuned versions of the ChatGPT model [15,73]. For example, in Study 3, the sequence in which we presented the transcripts in the prompts (actual painting followed by replica or vice versa) affected the phrasing of the final summary. Similarly, in Study 1, we discovered that the sentiment score output could change when we submitted the same texts in a different order. A possible explanation for this is that any minor variations in the prompt and output continue to propagate (or even reinforce) in subsequent summaries, and that ChatGPT lacks a self-correction mechanism allowing it to converge to a specific optimal response. This sensitivity, combined with the limited context length offered by ChatGPT, motivated our choice to process the texts in batches. The rationale behind using multiple batches or the bootstrapping method is the expectation that random noise will average out with enough repetitions of varied prompts, a principle resembling the self-consistency method [32]. In an alternative approach to Study 1, we submitted all 992 comments per AR interface simultaneously, using a newer model with an extended context length (gpt-3.5-turbo-16k). However, this approach yielded outputs of mediocre criterion validity, likely because of the single sentiment score given for the entire set of texts.

Another point of consideration pertains to the pipeline created from Study 3, which reduced the pairwise comparisons between transcripts of the original and replica paintings to a meta-summary. This process was found to be somewhat volatile and prone to errors. One significant challenge was the potential loss of vital information. For instance, depending on the prompt, ChatGPT might only highlight the difference between the two transcripts without indicating whether it is from Transcript 1 or 2. It might also produce output in inconsistent formats, such as narrative text versus bulleted statements. Furthermore, ChatGPT could generate over-generalized statements. An example is stating that participants closely examined the painting. Such a statement is likely applicable to both the original and the replica, offering little distinction between the two. As in Study 2, we also observed instances where specific observations from a few participants could become unduly prominent in subsequent summaries. The robustness check we applied—where the transcripts were submitted in two different sequences—was beneficial in this regard. We believe that an effective approach to creating meta-summaries involves retaining information that consistently emerges across various prompting approaches. This strategy aligns with the bootstrapping method and the self-consistency method previously discussed [32]. We further recommend involving a human researcher in the summarization pipeline to ensure ChatGPT's outputs are neither too generic nor overly detailed.

In summary, it can be posited that ChatGPT produces reproducible output, but this does not imply that reliance should be placed on a single output. Researchers are advised to report the precise version number of ChatGPT in their paper, so that other scholars may select the same model if necessary. Secondly, researchers are recommended to examine the robustness of the output, for example, through methods such as bootstrapping or by applying minor variations to the prompt.

Moreover, it must be recognized that, as indicated above, the autoregressive nature of ChatGPT means it is not especially proficient at tasks that require retaining multiple variables in memory, including counting [11]. For instance, in regard to Study 2, when trying to determine whether ChatGPT could perform a content analysis (akin to table 2, created by a human researcher), we discovered that ChatGPT did not provide particularly accurate output. Here, we tried various prompt strategies, such as simultaneously submitting the interviews from all participants (using gpt-3.5-turbo-16k) and directly prompting ChatGPT to create a table. We also attempted a more structured approach, where we presented all categories from table 2 to ChatGPT for each interview separately, prompting it to indicate whether or not the topic was addressed in the interview. In all cases, ChatGPT produced tables that were credible and fairly consistent with the narrative of the interview summary. However, the exact numbers (and the participants to whom these counts could be traced) were found to be insufficiently inaccurate and sensitive to the specific prompt. We believe these issues can be traced back to the aforementioned functioning of ChatGPT: it understands textual structures and can generate summaries of texts, but unlike humans, it is not capable of keeping counts in working memory required to make an overview table. It should be noted, however, that a human researcher cannot carry out this task alone either, but typically employs tools such as spreadsheet software or other forms of annotation or note-taking tools. In our case, the researcher used Microsoft Excel to perform the content analysis that ultimately resulted in table 2 (see supplementary material).

It should be further noted that ChatGPT's outputs also have limitations due to potential biases. These biases may arise from the fact that ChatGPT models have been trained on human-generated text and reinforcement learning from human feedback to better align with human values [74,75]. In particular, ChatGPT outputs could potentially contain biases toward political leanings [76–79]. These possible biases are unlikely to have affected the results of Study 1, because ChatGPT was tasked to provide numeric scores and not to generate new ideas. However, it may have affected the results of Study 2 to some extent, wherein it caught our attention that ChatGPT-4 devoted attention to individuals who are colourblind in the generated summary. The inclusion of colourblind or otherwise impaired road users in traffic indeed represents an important human value, and perhaps also one that a human researcher would have wanted to consider; however, strictly speaking, it was not something that was prominently featured in the 30 summaries and therefore did not emerge in the human content analysis. The challenge of alignment, that is, to what extent the output that large language models generate aligns with human values, is an ongoing research topic [80,81]. It can also be noted here that these values may be context-dependent, whereby in some cases, a researcher might want to receive a mechanistic output, such for a meta-analysis, and in other cases, they would prefer an output that takes into account more general human values such as safety and inclusiveness.

## 6. Conclusion

This work addressed the research question: To what extent does ChatGPT produce valid sentiment scores and summaries when applied to different forms of text data in HCI studies? We employed a hierarchical approach because ChatGPT has a limit to the number of tokens it can process. Specifically, we provided batches of text for it to summarize before producing a final score or summary. The results showed that (1) ChatGPT produced sentiment scores that correlated extremely highly with quantitative metrics, (2) produced meaningful summaries of interviews that aligned with content analysis, and (3) highlighted subtleties from think-aloud data that corresponded with a prior keyword count.

In conclusion, our findings suggest that ChatGPT is a valid tool for qualitative data analysis in HCI research. However, it is recommended to use ChatGPT as a complementary tool in conjunction with more quantitative analyses and critical human reasoning, areas in which the model currently shows limitations.

A strength of our study is that it examined various forms of validity across different types of texts, ranging from succinct textbox responses to patterns discernible in think-aloud data. Nevertheless, this work should be viewed as an initial step, and it is recommended that additional research, encompassing a more diverse range of text types and prompts, be undertaken. The rapidly evolving landscape of large language models, characterized by the increasing availability of a diversity of models, necessitates continual evaluation.

## Data Availability

The MATLAB scripts, as well as the ChatGPT inputs and outputs, are available in the following repository: https://doi.org/10.4121/21916017 [82].

## Footnotes

1 The number of outputs over which the sentiment score was calculated varied between 189 and 200, due to exceeding the token rate limit in certain instances, which resulted in no data being output in our loop.
